# Development and Performance Analysis of High-K Spacer-Induced Strained Si/SiGe Channel-Based Gate All Around FET for Thermal Effects

**DOI:** 10.3390/nano15231810

**Published:** 2025-11-29

**Authors:** Potaraju Yugender, Sneha Singh, Kuleen Kumar, Rudra Sankar Dhar, Alexey Y. Seteikin, Amit Banerjee, Ilia G. Samusev

**Affiliations:** 1 Department of Electronics and Communication Engineering, National Institute of Technology Mizoram, Chaltlang, Aizawl 796012, Mizoram, India; potaraju.ece.phd@nitmz.ac.in (P.Y.); dt23ec001@nitmz.ac.in (S.S.); 2 Department of Electronics and Communication Engineering, Malla Reddy (MR) (Deemed to be University), Hyderabad 500100, Telangana, India; 3 Department of Electronics and Communication Engineering, National Institute of Technology Puducherry, Thiruvettakudy, Karaikal 609609, Puducherry, India; kuleen.k@nitpy.ac.in; 4 Research and Education Center for Fundamental and Applied Photonics & Nanophotonics, Immanuel Kant Baltic Federal University, 236000 Kaliningrad, Russia; seteikin@mail.ru; 5 Microsystem Design Integration, Laboratory of Physics Department, Bidhan Chandra College, Asansol 713303, West Bengal, India

**Keywords:** gate stack, Si/SiGe channel, GAA FET, linearity and harmonics analysis

## Abstract

A Gate Stack GAA FET using SiGe with a 2 nm gate underlap encapsulating a high-k spacer has been created, explored, and evaluated for improved performance in radio frequency applications. The chip shows significant improvements in electrical and radio frequency analog performance because of the use of wrapped underlaps of high-k, which suppress parasitic capacitance and fringing field effects, to achieve a 192.52% boost in drain current and 98% reduction in I_OFF_ current, translating into better performance. This new device, as proposed, has demonstrated improved switching behavior with the ability to reduce subthreshold swing by about 11.24% and results in a better Ion/Ioff ratio over existing devices, while also maintaining efficient control over other SCEs, with it being well-suited for the implementation of high-performance and low-power CMOS circuits. In addition, linearity parameters like VIP2, VIP3, and IIP3 reflect improvements, with the device having lesser harmonic distortions (IMD3 and THD), therefore making it more appropriate for RF and analog circuit uses. These results point to the prospect of SiGe-based Gate Stack GAA FETs with a 2 nm gate underlap encircling a high-k spacer for low-power, high-speed applications in IoT and 5G/6G technologies toward building environmentally friendly and sustainable electronic solutions.

## 1. Introduction

With continuous development in semiconductor device technology, FET (Field-Effect Transistor) device dimensions are consistently scaled down [1,2,3]. The miniaturization has enabled higher device density, low power consumption, fast operation, and reduced costs [3]. However, as channel length reduced to the nanometer regime, the source-to-drain distance also decreased, the gate control over the channel weakened, and as a result, the transistor’s performance degraded. In addition to that, the leakage current also persists in the off condition of the transistor [4]. Therefore, to improve the controllability over the channel region, different architectures such as double gate (DG FET), Tri gate (TG) FET, FinFET and gate-all-around (GAA) FETs have been reported in the literature [5,6,7].

The FinFET introduced in 2011 [8] was the first three-dimensional structure to replace conventional Metal Oxide Field-Effect Transistors (MOSFETs). The FinFET is widely adopted for designing the analog circuit, analog to digital converter, and multiplier circuit [5,8,9,10].

The tunnel FET (TFET) is another emerging device in the nano regime. The low subthreshold in the range of less than 60 mv/dec can be obtained with high switching speed at value of 0.5 v and is well deserved in low power applications [11,12]. In addition, TFETs have been utilized in biosensors, semiconductors memories, and analog and digital circuits with minimal power dissipation [13,14].

The GAA FET is a multigate device that is composed of a nanowire in which the gate material is deposited overall to the channel region with circular, rectangular, or elliptical geometry, which is not possible in the FinFET device’s structure [15,16]. Therefore, better control over the channel region can be acquired as compared to the FinFET. Among all multigate FET devices, the GAA FET developed below 22 nm shows high immunity to short channel effects (SCEs) and better electrostatic coupling, which help to modulate the channel current accurately [16].

The DG FET with strain silicon developed by Kumar et al. [5] and the Triple-Fin Heterostructure-on-Insulator Fin Field-Effect Transistor developed by Saha et al. [17] shows enhancement in drive current, but are not suitable below a 10 nm channel length due to performance degradation. The induction of strain silicon technology with a high-k dielectric stack and a tri-layer silicon channel in a rectangular GAA FET can significantly enhance the performance parameters [17]. Therefore, below 10 nm, technology node devices need to be developed in GAA geometry with high-k materials and then they can be the best alternative to enhance performance.

Temperature changes have a significant impact on the electrical and thermal behavior of silicon-based gate-all-around Field-Effect Transistors, which is important for device performance, reliability, and scaling at advanced technology nodes [18,19,20,21,22]. As the temperature rises, phonon scattering in the silicon channel intensifies, resulting in a reduction in carrier mobility and, consequently, a decrease in the ON-state current. However, under moderate temperature increases, the generation of carriers improves, leading to an increase in OFF-state leakage currents and a corresponding decrease in the subthreshold slope. Different simulations and experiments indicate that temperature enhances the drive current (ON-state currents) due to increased carrier activity, but it also increases leakage in the OFF state. For example, a device that operates at room temperature will provide the optimum drive current. Cooler temperatures will help reduce power dissipation. As temperatures increase, the number of carriers that are thermally excited also increases, which in turn leads to increased leakage and diminished switch sharpness. Higher temperatures also degrade the subthreshold swing, requiring higher gate voltage to achieve specific current increases. Additionally, the threshold voltage typically decreases due to increased temperature, leading to early turn-on of the switch and increased static power consumption. Self-heating effects have become significant for nanoscale 3D architectures such as GAAFETs, where scaling the devices and stacking them will inhibit heat dissipation pathways.

Considering the above aspects of heterostructure channels, and with the inclusion of high-k material, a novel rectangular GAA FET device is developed here for the first time. The device is designed with a high-k spacer with a gate stack for enriching the device’s performance. Further in this paper, the device is analyzed at different temperatures with different gate stack configurations in order to find out the performance tradeoffs of different structures for different applications.

## 2. Device Structure and Methodology

To develop a more comprehensive gate-all-around FET, we designed three different devices: SiGe-based GAAFETs named A_1_, A_2_, and A_3_; SiGe-based GAAFETs with a gate stack (GS GAAFET) named B_1_, B_2_, and B_3_; and SiGe GS GAAFETs with a 2 nm gate underlap wrapping high-K spacer named C_1_, C_2_, and C_3_. Each device was simulated at different temperatures, 300 K, 350 K, and 400 K, for comparison and thorough analysis. These devices have a channel length of 10 nm, a source/drain length of 10 nm, and a Fin area of 7 × 7 nm^2^. The SiGe GAAFET features a tri-layer channel, which enhances performance by providing high hole mobility in the p-type channels and facilitating better strain engineering, resulting in a more power-efficient device with a high drive current. The SiGe gate stack GAAFET, includes a high-k dielectric that can help with gate leakage, interface quality, and compatibility with advanced engineering. The main aim is to optimize interface properties and control carrier mobility. Table 1 shows the dimensions of all the devices developed.

**Figure 1:**
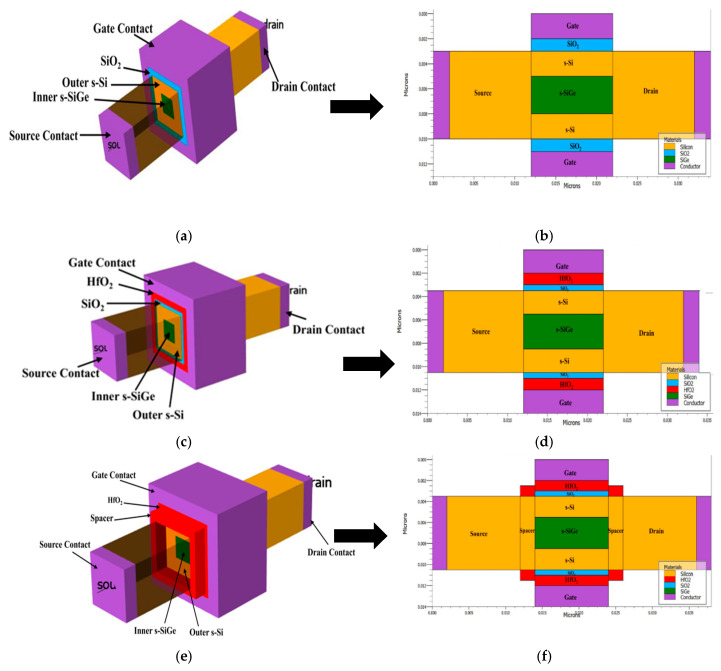
(**a**) Three-dimensional structure of device A1, (**b**) 2D X-Z cutline of device A1, (**c**) 3D structure of device B1, (**d**) 2D X-Z cutline of device B1, (**e**) 3D structure of device C1, and (**f**) 2D X-Z cutline of device C1.

Figure 1a shows the three-dimensional view of device A1 and Figure 1b shows the two-dimensional X-Z cross-section of device A1. Figure 1c shows the three-dimensional view of device B1 and Figure 1d shows the two-dimensional X-Z cross-section of device B1. Figure 1e shows the three-dimensional view of device C1 and Figure 1f shows the two-dimensional X-Z cross-section of device C1. Figure 1f illustrates the 2D cross-sectional schematic of the proposed wrapped underlap-strained Si/SiGe channel heterostructure GAA FET. Unlike conventional spacer technologies, where the spacer material remains laterally adjacent to the gate without direct overlap, the wrapped underlap configuration allows the gate’s oxide material to extend over the region to reach the source/drain forming a high-k spacer layer, precluding fringing field-coupling effects across the region. This design enhances the gate-to-channel coupling and modulates the potential in the underlapped segment, thereby improving subthreshold behavior and reducing drain-induced barrier lowering (DIBL). The labeled schematic clearly identifies all critical regions including the gate, spacer, tri-layered strained Si/SiGe heterostructure channel, source/drain, and the wrapped underlap portion to ensure structural clarity and reproducibility of the nanostructured device.

Inserting SiO_2_ and HfO_2_ in the channel region of a gate stack GAAFET device yields many benefits, as they are the very crucial materials for the gate dielectrics in the scaling of GAAFET devices. This improves carrier mobility, interface quality, and reduces gate leakage, as the HfO_2_ high-k dielectric has a much higher dielectric constant than SiO_2_. This allows the gate’s oxide to be thick, reducing tunneling leakage and maintaining a low equivalent oxide thickness (EOT). The gate stack controls the gate and minimizes the short-channel effects, maintains low leakage, and high capacitance. The SiO_2_/HfO_2_ stack improves overall device reliability and prevents threshold voltage shifts. Devices C_1_, C_2_, and C_3_ are designed with an underlap length of 2 nm, as illustrated in the sectorial view in Figure 1e, which is a 2D X-Z cutline of device C_1_.

**Figure 2:**
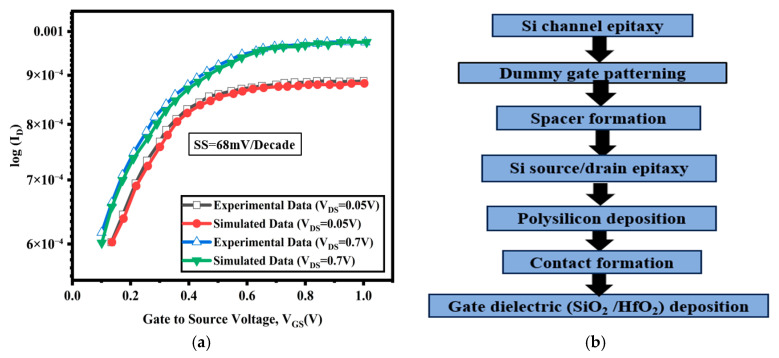
(**a**) Electrical parameter fitting for calibration to the experimental data and the physical parameters of the device [21] with I_D_-V_GS_ logarithmic graph, where V_DS_ represents drain voltage; (**b**) fabrication process flow of Si-based GAA FET.

The results from the Silvaco Atlas simulator (The equipment ‘Silvaco Atlas simulator’ was sourced from Silvaco International, Santa Clara, CA, USA, as cited in the user manual [23]) are validated by comparing them to the ID–VGS graph found in the literature, which contains simulated data for device H (Ritzenthaler et al. [21]). The ID–VGS characteristics of the configuration suggested by Ritzenthaler (an experimentally developed two-stacked Si GAA FET with a gate length of 26 nm) were produced at VDS values of 0.7 V and 0.05 V, as presented, which align closely with the results simulated using Silvaco by integrating the Hansch quantum model and the Lombardi mobility model, as illustrated in Figure 2a. Consequently, device H acts as a reliable reference for calibrating the electrical characteristics of the new GAA devices created in this work. To ensure the accuracy and experimental relevance of the proposed device model, the simulation parameters for the strained Si/SiGe channel GAA FET were meticulously calibrated against experimental data from Si nanowire (NW) CGAA FETs [21]. Figure 2a illustrates a close match between the simulated and experimental transfer characteristics, confirming the robustness of the calibrated TCAD model. Furthermore, all the extracted parameters including drain current, transconductance, linearity, and harmonic distortion exhibit excellent correlation with previously reported experimental results [21] and align well with the IRDS 2025 technology roadmap. This validation establishes strong confidence in the physical accuracy of the simulation and supports the predictive capability of the proposed device configuration. The application of strain technology in the channel area of SiGe-based GAAFETs creates bandgap narrowing through strain engineering, The device generates carrier accumulation through ballistic transport inside the channel, which is considered in the overall analysis of the strained-channel SiGe-based GAAFET. The new device includes a Type-II heterostructure that allows for both carrier confinement and strain effects, forming a quantum well barrier in that area. As a result, a cylindrical nanosystem with a strained channel is made, which supports ballistic transport through a very thin barrier layer. This widening leads to an increase in the bandgap energy between the allowed energy levels in that thin region, which ultimately counteracts the tensile strain and may reduce SCEs while improving performance. The developed SiGe-based GAAFET device appears to surpass the IRDS 2025 target for the 2 nm technology node device by using strain channel engineering, a stacked high-k gate, and a high-k spacer with gate underlap. The fabrication of a 10 nm strained n-channel SiGe-based GAAFET with a stacked high-k gate involves three main steps: meshing, material filling, and doping. Based on the materials used in different parts of the produced meshes, the device’s structure is divided into separate regions. Silicon is used as the dopant, silicon oxide is the interface layer, high-k (HFO_2_) is used as the spacer and gate dielectric, and polysilicon serves as the gate contact. Figure 2b shows the possible process flow for making the device. Epitaxial growth is used to create the channel region, forming a two-layer stressed nanosystem. The channel has a multilayer structure where the outer layer is silicon and the inner layer is Si_1−x_Ge_x_ with a mole fraction of x = 0.4, and it is grown to fill the inner cylindrical space. The strained hetero-channel zone is formed by placing a top Si layer over the inner SiGe part, creating a quantum well barrier nanosystem channel.

The devices simulated here employ Silvaco TCAD tools [23]. Physical models such as bandgap narrowing, Auger recombination, Shockley–Read–Hall (SRH) recombination method, Concentration-Dependent Carrier Lifetimes model for fixed carrier lifetimes in SRH recombination due to impurity concentration, Hansch quantum effects approximation model for N-channel MOS devices, and Fermi statistics for electrons and holes are employed in parallel in this device analysis. The Newton and Gummel methods are both based on numerical simulation-based calculations. SILVACO applies numerical iteration of the Newton and Gummel methods to recombine characteristics and to solve differential equations, enabling advanced analysis of innovative devices developed for the future. Silvaco TCAD device simulations offer comprehensive models for temperature-dependent behavior to accurately mimic the real-world operation of semiconductors. The models are of extreme importance in predicting how the properties of devices vary with changes in temperature for silicon-based technology, including GAAFETs. We used the Bandgap Narrowing Model when semiconductors have a reducing bandgap as temperature increases. Silvaco ATLAS and Sentaurus both apply a similar physical model. The quantum well confinement enhances carrier transport by suppressing intervalley scattering and increasing channel inversion efficiency under gate bias. The strain-induced modification of the bandgap and carrier distribution further contributes to reduced subthreshold swing and enhanced on-state current. These effects collectively demonstrate the superior electrostatic and transport characteristics of the strained Si/SiGe heterostructure GAAFET compared to conventional unstrained Si-based architectures. Silvaco fixes the silicon bandgap at 300 K to 1.08 eV. Rising temperatures cause a decrease in Eg, which in turn provides a greater intrinsic carrier concentration, thus impacting the threshold voltage and leakage current. Mobility (μ) is affected by phonon (lattice) scattering at elevated temperatures and by impurity scattering at reduced temperatures. Enhanced models distinguish between lattice and impurity scattering contributions for improved accuracy at both low and high temperatures. The coefficients for impact ionization are temperature-dependent, which is vital for simulations of breakdown voltage (for instance, Valdinoci’s model of temperature-dependent impact ionization). Bias temperature instability (BTI), particularly negative BTI in pMOSFETs, is significantly influenced by temperature and can be modeled using approaches like MPFAT (Multi-Phonon Field-Assisted Trapping) in Silvaco. The developed 10 nm SiGe-based GS GAA FETs with a 2 nm gate underlap wrapping high-K spacer device are then compared for improved performance and examined for quantum effect analysis at the nanoscale to be used further for faster device-switching operations.

## 3. Results and Discussion

### 3.1. DC Analysis

**Figure 3:**
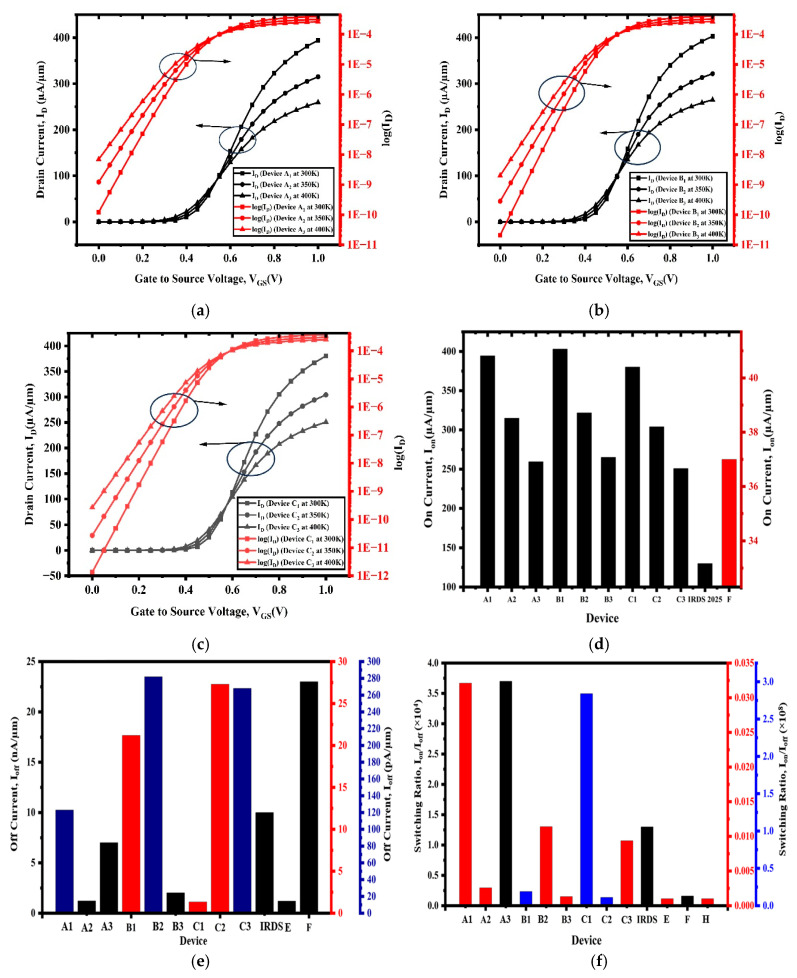

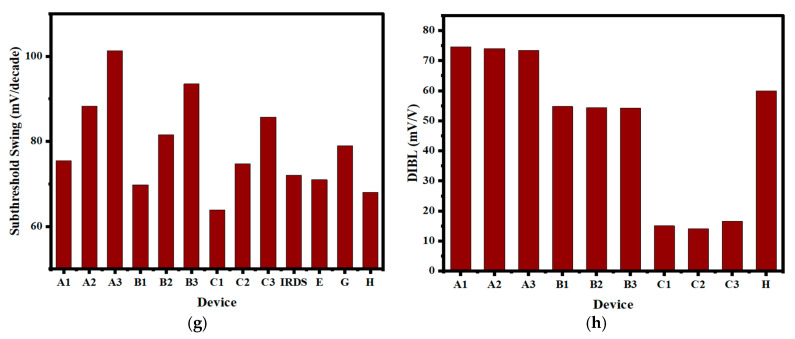
(**a**) I_D_ vs. V_Gs_ graph in both linear and logarithmic scales of device A_1_, A_2_, and A_3_, (**b**) I_D_ vs. V_GS_ graph in both linear and logarithmic scales of device B_1_, B_2_, and B_3_, (**c**) I_D_ vs. V_GS_ graph in both linear and logarithmic scales of device C_1_, C_2_ and C_3_, (**d**) comparative analysis of ON current I_on_ among all devices, (**e**) comparative analysis of OFF current I_OFF_ among all devices, (**f**) comparative analysis of I_ON_/I_OFF_ ratio among all devices, (**g**) comparative analysis of subthreshold Swing among all devices, and (**h**) comparative analysis of DIBL among all devices.

Figure 3a represents the drain current ID versus the gate-to-source voltage (VGS) characteristics of device A at different temperatures: 300 K, 350 K, and 400 K. The graph has two Y axes, which display both the linear and logarithmic drain current with the voltage V_GS_ ranging from 0 to 1 V.

The graph provides a comprehensive understanding of the electrical performance of device A at different temperatures of a SiGe-based GAAFET. The solid curves represent the linear drain current, I_D_, at different temperatures (300 K, 350 K, and 400 K), while the logarithmic drain current I_D_ curves show the subthreshold and leakage regimes with enhanced values. When the gate voltage, V_GS_, is high, the drain current, I_D_, increases sharply, indicating effective gate control and strong channel formation. When the temperature increases from 300 K to 400 K, we observed two primary effects: firstly, it increases the drain current, I_D_, as the temperature rises; secondly, it enhances carrier mobility and reduces the threshold voltage. Additionally, the subthreshold and OFF state currents emphasize the rise in leakage current in the subthreshold region, which impacts the static power consumption and the overall reliability. The data display distinct trends and overlapping operating points, highlighted on the plot to denote areas of interest where temperature dependence is most significant. The low-temperature profile indicates reduced subthreshold swing and limited leakage, whereas the high-temperature range exhibits increased off-state currents. The figure shows that the SiGe-based GAAFET device maintains a strong ON-state drive current across three different temperatures; however, the design is affected by rising OFF-state and leakage currents at high temperatures. The graphical representation shows the importance of power management and the need to realize high-performance, low-leakage transistors for nanoelectronics.

Figure 3b shows the transfer characteristics of a SiGe-based gate stack GAAFET (device B) plotted between drain current I_D_ versus gate-to-source voltage V_GS_ at 300 K, 350 K, and 400 K. It represents both the linear scale and logarithmic scale of drain currents. The device’s performance offers a comprehensive view across its ON and OFF regimes as the gate’s bias increases from 0 to 1 V. The graph evaluates the electrical behavior of the Si-Ge-based gate stack GAAFET device (device B). The black curves show the linear drain current I_D_ values at 300 K, 350 K, and 400 K, while the red curves show the logarithmic drain current I_D_ values, which provide a clear insight into both the ON-state drive and subthreshold/OFF-state leakage current behavior. When the temperature rises from 300 K to 400 K, the drain current increases for the given V_GS_ in the ON-state. At high temperatures, the behavior is due to increased carrier generation and a reduced threshold voltage. From the logarithmic curves at low values of V_GS_, it is evident that the leakage current increases with temperature, which is beneficial for low-power applications. The increased subthreshold slopes and punch-through the leakage-assisted carrier injection. At low temperatures, the device maintains a robust ON current with good subthreshold suppression and a strong gate electrostatic control. From the characterization analysis of transfer characteristics, it is clear that the gate stack GAAFET device has high ON-state drive current and very good subthreshold swing at room temperature, although there is an expected increase in leakage and threshold voltage roll-off behavior at elevated temperatures. The outcome emphasizes the important role of interface engineering, gate dielectric selection, and channel design to preserve performance and power efficiency across a variety of use environments.

Therefore, detailed temperature-dependent characterization is essential to benchmark state-of-the-art GAAFETs, especially when used in ultra-scaled nodes, where thermal effects, leakage, and variability start to dominate device behavior. The result supports the device’s concept for next-generation low-power, high-performance CMOS logic, while demonstrating the tradeoffs of the engineering.

Figure 3c represents the drain current I_D_ versus gate-to-source voltage V_GS_ characteristics of device C at different temperatures of 300 K, 350 K, and 400 K. The graph presents the transfer characteristics of a SiGe-based gate stack GAAFET with gate underlap (device C) by plotting the graph between the drain current against the gate-to-source voltage V_GS_ at three different temperatures (300 K, 350 K, and 400 K). It displays the graph in both linear and logarithmic scales, illustrating the device’s operation across the voltage range from 0 to 1 V. It explores the effect of temperature on the electrical parameters of device C with a gate underlap. The curves give a detailed conception of the ON-state drive and subthreshold leakage. The observations from the graph show that as the temperature increases, the device’s temperature dependence results in an increase in drain current above the threshold voltage, a reduction in specified carrier mobility, and a decrease in threshold voltage. At low V_GS_ voltage, the leakage current increases with temperature, which helps suppress SCEs. The device’s operation changes rapidly in response to temperature fluctuations, demonstrating its electrostatic control and sensitivity. The performance of device C at 300 K displays powerful gate control, low leakage, and a sharp subthreshold swing, validating the effectiveness of the gate underlap device. At 400 K, the leakage and threshold voltage roll off, emphasizing the importance of carefully engineering the interface, as well as the spacer layer. The ON/OFF ratio remains large, allowing for reliable switching time and low off-state power consumption, which is typical of gate stack GAAFETs with underlaps for use in next-generation devices. The transfer curves, dependent on temperature, depict the impact of gate underlaps on GAAFET architectures in terms of ON current, threshold control, and leakage. The data in this figure clearly demonstrate the use of gate underlaps to reduce SCEs while sustaining high ON currents and maintaining leakage at nominal operating conditions. Gate stack GAAFETs with underlaps are viable candidates in ultra-scaled, low-power, and high-performance logic circuits and will compete as one of the best transistors in future technology nodes of semiconductor technology.

It is observed that the drain current I_ON_ of devices B_1_ and C_1_ achieves higher values compared to the other devices. Figure 3d shows the drain current values of all the devices. Both devices, B_1_ and C_1_, overcome the benchmark against the IRDS 2025 and reference device F, which indicates superior electrostatic control and carrier mobility. This figure indicates that various superior gate stack GAAFET configurations well surpass the current IRDS 2025 ON current specifications, verifying the performance of better materials and geometric design in emerging transistor developments. The comparison emphasizes the importance of continued optimization of channel structure and gate dielectric selection to surpass industry standards. The inclusion of device F and IRDS 2025 as standards further emphasizes the achievements and current challenges in pushing high-performance logic transistors. Figure 3e shows the comparative analysis of the OFF-state leakage currents of all the devices, and it is observed that devices B_2_, C_1_, and C_2_ exhibit the highest OFF-state leakage currents, which have the potential for improved ON-state performance. Devices A_2_, A_3_, B_3_, and C_3_ show low OFF-state leakage currents, which point to superior leakage control. Devices A_1_, B_1_, and F have a moderate leakage current. This comparative analysis demonstrates the effectiveness of various gate stack and channel engineering techniques for ultra-scaled GAAFETs. Achieving an optimal OFF-state current while maintaining good ON-state performance is essential for future technology nodes, where reducing static power is as crucial as increasing speed. The results present promising designs but highlight the need for continued innovation to meet tight leakage targets set by global standards such as the IRDS. Figure 3f presents a comparative analysis of the switching ratio among all devices. Devices A_1_, A_2_, and C_1_ show the highest switching ratio with greater electrostatic gate and effective leakage suppression. These devices are capable of ensuring lower static power and a high I_ON_/I_OFF_ ratio, which are crucial for high-performance and energy-efficient devices. Figure 3g presents the comparative analysis of subthreshold swing among all the devices. We observe that devices A_1_, B_1_, and C_1_ have low values of SS, which have better control over the channel and switch more efficiently. The values are also within the IRDS limit. Figure 3h presents the comparative analysis of DIBL among all the devices. From the figure, devices C_1_, C_2_, and C_3_ have the lower DIBL values, which means the threshold voltage of the devices is more consistent, minimizing the short channel effects, and improving the device’s stability.

The electrical characteristics of the proposed strained Si/SiGe channel GAA FET (device C_1_) were obtained using Silvaco TCAD simulations. The simulation parameters were benchmarked and calibrated against the experimentally fabricated Si nanowire (Si NW) cylindrical GAA FET reported in Ref. [21], as already discussed previously in Section 2 and shown in Figure 2. Furthermore, it is validated with IRDS 2025 technology projections to ensure physical consistency. Figure 3 presents the comparative electrical analysis among the developed device structures (A_1_–A_3_, B_1_–B_3_, and C_1_–C_3_), the fabricated experimental device H [21], and the simulated existing devices [11,12,13,14,15]. The results clearly demonstrate that the proposed device C_1_ exhibits superior electrical performance, achieving a higher I_ON_, lower off current, a higher switching ratio, and lower SS and DIBL compared to all other developed, existing simulation and fabricated devices as shown in Figure 3 and summarized in Table 2. The rigorous calibration, benchmarking, and validation process ensures that the simulation’s outcomes are both physically realistic and experimentally consistent, confirming that the proposed structure represents an optimized design for next-generation nanoscale GAA FET technologies.

**Figure 4:**
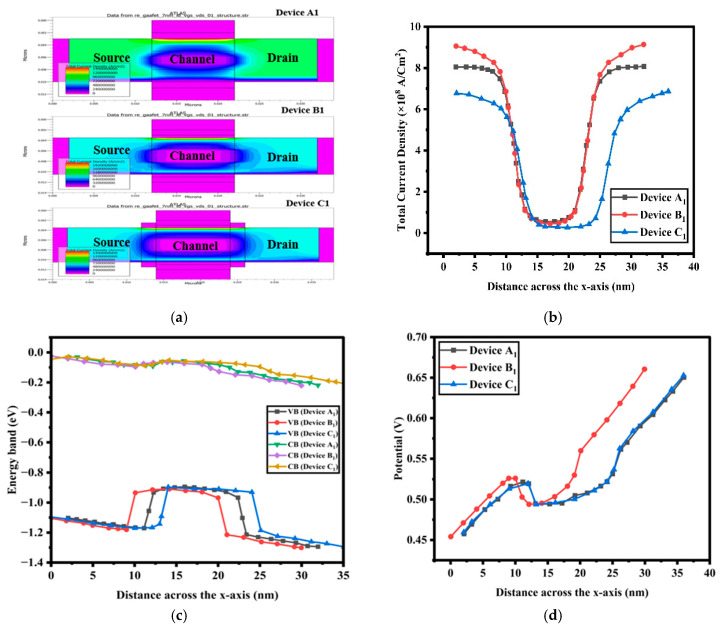
(**a**) Contour diagram of total current density of devices A_1_, B_1_, and C_1_, (**b**) graphical presentation of total current density of devices A_1_, B_1_, and C_1_, (**c**) energy band diagram of devices A_1_, B_1_, and C_1_, and (**d**) graph of potential vs. distance the *x*-axis for three devices A_1_, B_1_, and C_1_.

From Table 2, one can see that device C_1_ is not only in a position to satisfy but surpass the specifications that have been placed on IRDS 2025 using its 2 nm node technology. To explore this further, we venture into a detailed investigation of device C_1_. Figure 4a shows the contour diagram of total current density v of devices A_1_, B_1_, and C_1_. Large current density changes are seen at the source–channel and drain–channel interfaces of the outer st-Si layer. The corners of these interfaces have a high current density because carrier movement is limited there, while the average current density remains fairly uniform across the outer st-Si layer. The confinement of electronic carriers in the channel is caused by the combined effect of Type-II band alignment and the stacked high-k dielectric in the st-Si channel device. Since SiGe is very sensitive to hole-carrier motion, the outermost st-Si layer benefits from better electron transport through ballistic motion. It also needs to be highlighted that high-k spacers increase capacitance in the fringing fields, and thereby current density is enormously increased at the source–channel as well as drain–-channel interface.

Figure 4b presents the current density distributions generated using the Silvaco TCAD tool. The source–channel and drain–channel interfaces display the contours of the current density of devices A_1_, B_1_, and C_1_. Table 2 presents the electrical parameters of all of the devices. Among these devices, C_1_ stands out as the best, offering superior performance for CMOS and RF applications. It features a high ON current, low subthreshold swing, improved switching, and strong leakage suppression.

Figure 4c shows the CB and VB energy levels of devices A_1_, B_1_, and C_1_, clearly demonstrating the superior performance of device C_1_ over the others. The conduction and valence band energies in device C_1_ exhibit increased stability and improved characteristics, reflecting its enhanced design and performance. The addition of a 2 nm high-k HfO_2_ underlap enhances the lower conduction band edge, improving electron confinement and reducing leakage currents, thereby improving the on-state performance and power efficiency of device C_1_. SiGe-based GS GAA FETs are of a wide band gap, and this contributes to enhancing the thermal stability and reliability of the valence band energy at high temperatures. This is due to the improved heat dissipation associated with increased carrier confinement and enhanced transport characteristics, rendering the device stable at high temperatures. The proposed device utilizes a strained Si/SiGe channel heterostructure, where a thin tensile-strained Si layer is epitaxially grown on a relaxed Si_1−x_Ge_x_ buffer (x = 0.4). The lattice mismatch induces biaxial tensile strain in the Si channel, leading to band splitting in the conduction band valleys and an effective reduction in electron effective mass, thereby improving mobility. Figure 4c presents the calculated energy band diagram of the Si/SiGe heterostructure, showing a Type-II band alignment in which the conduction band minima of the strained Si layer lie below that of strained SiGe, forming a quantum well-barrier nanosystem that confines electrons within the strained Si layer. Figure 4d indicates the possible profiles along the channel from the drain to source for devices A_1_, B_1_, and C_1_ under operation. The red color indicates the higher potential values. Throughout the analysis, device C_1_ exhibits a greater surface potential than devices B_1_ and A_1_. This phenomenon is most apparent close to the drain end, where electrostatic potentials increase and the DIBL decreases. This reduction in DIBL indicates that device C_1_ is able to exercise more control over the channel, resulting in superior overall performance, reliability, and higher switching speed.

### 3.2. Linearity Analysis

**Figure 5:**
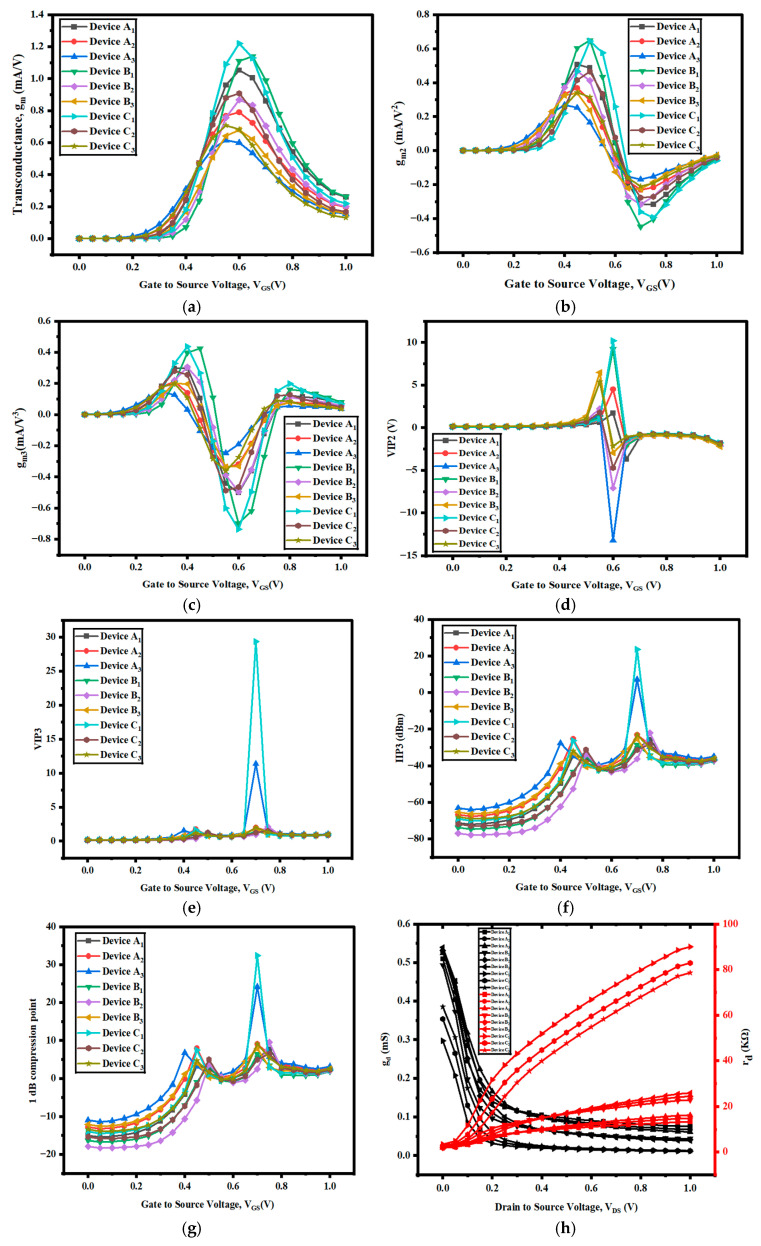
Variation in (**a**) g_m_ vs. V_GS_, (**b**) g_m2_ vs. V_GS_, (**c**) g_m2_ vs. V_GS_, (**d**) VIP2 vs. V_GS_, (**e**) VIP3 vs. V_GS_, (**f**) IIP3 vs. V_GS_, (**g**) 1 dB compression point vs. V_GS_, and (**h**) g_d_ and R_out_ vs. V_DS_ for all developed devices.

For effective communication in RF applications, the system must have a high degree of linearity. This entails enhancing the signal-to-noise ratio and minimizing harmonic distortion, which are the most important elements. According to [24,25,26,27], transistor nonlinear characteristics are mostly brought about by higher levels of gm2 and gm3 compared to gm. These are important in evaluating the linearity of the device, especially the second-order and third-order transconductances, and are graphed versus VGS for each of the three devices in Figure 5a–c. Using a high-k underlap spacer within a semiconductor-on-insulator technology lowers the transconductance of the second and third order relative to gm. To evaluate linearity and the device’s quality for RF applications, VIP2, VIP3, IIP3, IMD3, and 1 dB, gain compression point measurements are made for each device. Among all the devices, C_1_ has the best transconductance value of 1.13 mA/V, which is the highest value of all the devices, as shown in Figure 5a and Table 3.

Figure 5b,c graph the higher-order transconductances, the second- and third- order, against V_GS_ for each of the three devices. Second- and third-order transconductance decreases compared to gm are observed when using a high-k underlap spacer semiconductor-on-insulator technology (in device I), to evaluate the device’s performance and establish its suitability for RF applications, and to further the linearity analysis. For devices A, B, and C, VIP fluctuations for various values of V_GS_ in the second and third orders are shown in Figure 5d and Figure 5e, respectively. Compared to devices B_1_ and A_1_, device C_1_ shows larger values for both VIP_2_ and VIP_3_. Higher VIP values indicate greater linearity and better suppression of distortion, making the device suitable for analog and RF applications. Higher gate control and reduced short-channel effects in GAAFETs help in maximizing VIP to support high-frequency operation and robust analog signal integrity. The determination or measurement of VIP is performed through large-signal simulations and harmonic or intermodulation distortion analysis, and is optimized by varying gate stack materials, channel geometry, and biasing conditions. The second and third orders are VIP_2_ and VIP_3,_ whose values among all devices C_1_ are higher. When one conducts a test of linearity in radio frequency circuits with FinFET technology, the third-order input intercept point (IIP3) is the primary measurement. It indicates the power level at which the third-order modulation distortion product begins to be evident and decreases the linearity of the RF system. The IIP3 is greater when utilizing high-k semiconductors on an insulator, as illustrated in Figure 5f. IIP3 is the theoretical input power at which the amplitude of the third-order intermodulation distortion (IMD3) products is equal to the amplitude of the fundamental signal. It measures the linearity of a device when processing multi-tone signals. An increase in the value of IIP3 means that the device can process more powerful signals before experiencing significant nonlinear distortion. Device C_1_ has a higher value of 23.60 dBm, which is the standout among all the devices. The 1 dB compression point in the GAAFET (gate-all-around FET) refers to the input or output power level at which the device’s gain drops by 1 dB from its ideal small-signal linear gain. Figure 5g shows that the 1 dB compression point of the high-k spacer device C_1_ is larger than those of other devices. The former is due to the greater value of gm in device C_1_. The point of 1 dB compression, which represents the power level at which nonlinearity causes the gain of the device to fall by 1 dB, is crucial in radio frequency applications. Figure 5h shows the g_d_ and R_d_ vs. V_DS_ for all developed devices. The output conductance, g_d_, of device C_1_ is minimum, and the output resistance, R_d_, is a higher value, as shown in Table 3.

### 3.3. Harmonic Distortion

Rodrigo Trevisoli Doria [24] reports an evaluation of the DG GC transistors’ performance based on the GAA device structure, from the distortion point of view. Harmonic distortion (HD) is a term used to describe the inherently nonlinear nature of the output current of transistors, resulting in the presence of signals with frequencies other than those of the input signal. Thus, overall HD (THD) and third-order HD (HD3) will be taken as significant performance measures. Second-order HD (HD2) will not be discussed because this measure is relatively similar to THD in the considered operational range [25]. Distortion has been assessed employing the integral function method (IFM), which enables distortion extraction from DC measurements without AC characterization, as opposed to Fourier analysis-based methods [26], for instance. The linearity performance of GC GAA will be compared with that of conventional GAA transistors used in the saturation regime. In the study, the SOI MOSFET is used as a single-transistor amplifier, and HD is evaluated for different LLD/L ratios. The effect of diminishing channel length on linearity will also be considered. Long channel MOSFETs are selected for the best analog performance when used in baseband applications. In GAAFETs, harmonic distortion parameters are given in dBm. HD2 quantifies the nonlinear behavior of the device, producing signals at twice the fundamental frequency, which can interfere with wanted signals in analog/RF circuits. Third-order harmonics are especially challenging since they are closer to the fundamental frequency and can intermodulate with other signals. This is the sum power of the harmonics (usually the second to fifth harmonics) with reference to the fundamental frequency. THD is a measure of the overall nonlinear distortion, and devices with a lower THD (more negative dBm) are said to have more fidelity. Distortion measurement metrics such as HD2, HD3, THD, and IMD3 are critical for evaluating the linearity and signal integrity of GAAFET devices in RF and analog applications.

The increase in distortion is due to the nonlinear characteristic of equipment employed in analog and radio frequency applications, which is a main concern. Distortion degrades the signal by adding unwanted components that belong to a group other than the right frequency group. Therefore, we try to reduce distortion in the output of linear amplifiers as far as possible. To achieve this, the IFM (integral function method) will be employed to quantify distortion parameters through DC measurements instead of AC measurements. This is performed to establish the numerical interlinkages between different distortion measurements, like HD2 (second-order harmonic distortion), HD3 (third-order harmonic distortion), and THD (total harmonic distortion).

**Figure 6:**
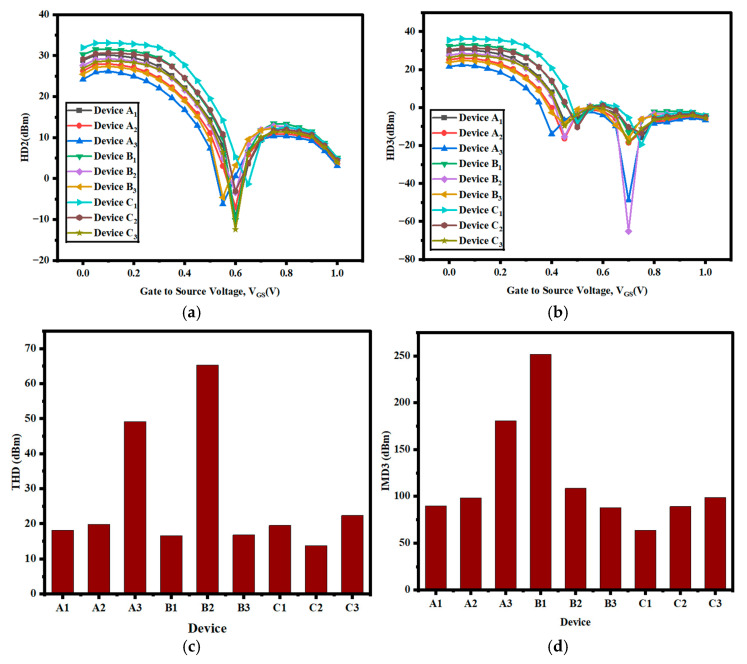
(**a**) HD2 vs. V_GS_, (**b**) HD3 vs. V_GS_, (**c**) bar graph of THD among all developed devices, and (**d**) comparative analysis of IMD3 and all developed devices.

Values of second- and third-order harmonic distortion and total harmonic distortion (THD) are maintained as close to zero as possible to minimize distortion during the device’s operation. The variation in HD2 is given in Figure 6a, whereas variations in HD3 and THD with V_GS_ are given in Figure 6b, c. The reduction in gm2 and gm3 reduces the diminution in transconductance, thus producing low levels of distortion. Consequently, device C_1_’s performance is better since its distortion characteristics are greatly improved by the lower second- and third-order transconductance. In order to enhance the linearity of the devices, the third-order intermodulation distortion (IMD_3_) ought to be kept at a minimum. For all the compared configurations, the curve of IMD_3_ versus VGS is presented in Figure 6d. For lower values of V_GS_, IMD_3_ rises due to the decrease in VIP3, which results in an increase in gm3. In contrast, at elevated V_GS_, the IMD_3_ reduction is attributed to an increased third-order voltage interception point, which reduces gm_3_ for device C_1_. It is worth noting that the present device has an IMD_3_ of approximately 5 dBm. Table 4 illustrates that the current distortion for device C_1_ is significantly lower than that of devices A_1_ and B_1_. From Table 4, we can conclude that device C_1_ is the best device, with HD2 (dBm), HD3 (dBm), THD (dBm), and IMD3 (dBm) values of −1.345, 19.47, 19.5164, and 63.80899, respectively.

To ensure the physical accuracy and validation of the simulation framework, the proposed device, C_1_, was compared with the fabricated and experimental device, H [21] (Si Nanowire Cylindrical GAA FET). Furthermore, the developed device variants (A_1_–A_3_, B_1_–B_3_, and C_2_–C_3_) were also analyzed under varying operating temperatures (300 K, 350 K, and 400 K) to examine thermal and electrical stability. The comparative results, summarized in Table 2, Table 3 and Table 4, clearly show that device C_1_ exhibits superior performance over all the newly developed devices, simulated devices [18,19,20,28,29], and the fabricated device [21]. The results further confirm that the proposed structure maintains excellent current drivability, subthreshold behavior, and scalability across a wide thermal range, ensuring strong correlation with experimental trends and IRDS 2025 projections.

The electrical, linearity, and harmonic analyses reveal that device C_1_ exhibits the best overall performance among all developed structures (A_1_–A_3_, B_1_–B_3_, and C_2_–C_3_) under varying temperature conditions (300 K, 350 K, and 400 K), as well as in comparison with the fabricated experimental device, H [21], and previously simulated devices [18,19,20,28,29]. As presented in Table 2, Table 3 and Table 4, device C_1_ demonstrates a 284.12% higher switching ratio compared to the existing fabricated device H [21], along with a 12.6% higher g_m1_, 80% lower g_m2_, 81.2% lower g_m3_, 75% higher VIP2, and 130% higher VIP3 compared to device A_1_. Furthermore, the THD value is 89.02% lower than that of the existing device [29], confirming the superior linearity and RF performance of the proposed C_1_ device.

## 4. Conclusions

The SiGe-based gate stack GAA FET with a 2 nm gate-underlap wrapping a high-k spacer (device C_1_) is developed here, and a comprehensive temperature analysis is performed at 300 K, 350 K, and 400 K temperatures. Device C_1_ is analyzed and compared with the electrical parameters of existing devices, namely devices E, F, G, and H. With respect to switching speed, leakage current (Ioff), ON current, subthreshold swing, and DIBL, Device C_1_ performs better than the present devices. The strained channel with st-Si, st-SiGe, and st-Si, combined with the SiGe-based gate stack GAA FET and a 2 nm gate underlap wrapped with a high-k spacer, enhances carrier mobility by means of ballistic transport and quantum carrier confinement in the channel. Device C_1_ demonstrates a significant improvement in the Ion/Ioff ratio with an ON current increase of 192.52% and an OFF current reduction of 98% from the IRDS 2025 roadmap. Device C_1_ demonstrated a significant improvement, with a 7.62% increase in transconductance. While the device had much improved linearity, the second- and third-order transconductance fell by 27.47% and 46.80% in comparison to device A1. VIP_2_ and VIP_3_ rose by 494% and 1870%, respectively. The 1 dB compression point and IIP_3_ fell significantly, which really indicates that the device has improved linearity compared to device A1. Also, IMD_3_, harmonic distortion, HD_2_, HD_3_, and THD all reduced, indicating less distortion compared to other devices. This makes device C_1_ a better option since it has more efficient carrier transport due to the high-k wrapped underlaps and the channel.

## Figures and Tables

**Table 1:** All developed device dimensions.

Device	Device Specification	Channel Length, L_Ch_ (nm)	Source/Drain Length, L_S/D_ (nm)	Fin Area (H_fin_ × W_fin_) (nm^2^)	Gate Underlap/High-K Spacer, L_un_/L_sp_ (nm)
A_1_ to A_3_	SiGe-based GAA FET at 300 K, 350 K, and 400 K	10	10	7 × 7	NA
B_1_ to B_3_	SiGe-based GS GAA FET at 300 K, 350 K, and 400 K	10	10	7 × 7	NA
C_1_ to C_3_	SiGe-based GS GAA FET with 2 nm gate underlap wrapping high-K spacer at 300 K, 350 K, and 400 K	10	10	7 × 7	2

**Table 2:** Electrical parameters of all developed and existing devices. Significant values are in bold.

Device	Vth (V)	I_on_ (µA/µm)	I_off_ (A/µm)	I_on_/I_off_	SS (mV/Decade)	DIBL (mV/V)
A_1_	0.35	394.462	1.23 × 10^−10^	3.21 × 10^6^	75.44	74.58
A_2_	0.32	314.981	1.22 × 10^−09^	2.58 × 10^5^	88.27	74.04
A_3_	0.29	259.522	7.01 × 10^−09^	3.70 × 10^4^	101.33	73.47
B_1_	0.37	403.139	2.12 × 10^−11^	1.91 × 10^7^	69.8	54.76
B_2_	0.34	321.833	2.82 × 10^−10^	1.14 × 10^6^	81.58	54.37
B_3_	0.31	265.097	2.02 × 10^−09^	1.31 × 10^5^	93.51	54.2275
**C_1_**	**0.40**	**380.275**	**1.34 × 10^−12^**	**2.84 × 10^8^**	**63.91**	**15.06**
C_2_	0.37	304.098	2.73 × 10^−11^	1.11 × 10^7^	74.76	14.1
C_3_	0.34	250.9	2.68 × 10^−10^	9.37 × 10^5^	85.71	16.6
IRDS 2025	Not Given	130	10 × 10^−9^	1.3 × 10^4^	72	Not Given
E [18]	Not Given	120	1.2 × 10^−9^	1 × 10^5^	71	Not Given
F [19]	Not Given	37	23 × 10^−9^	0.16 × 10^4^	Not Given	Not Given
G [20]	Not Given	Not Given	5.5 × 10^−9^	Not Given	79	Not Given
H [21]	Not Given	1 × 10^3^	10 × 10^−9^	1 × 10^5^	68	60

**Table 3:** Linearity analysis of the developed devices. Significant values are in bold.

Device	g_m_ (mA/V)	g_m2_ (mA/V^2^)	g_m3_ (mA/V^3^)	VIP2 (V)	VIP3 (V)	IIP3 (dBm)	1 dB Compression Point	G_d_ (mS)	R_d_ (KΩ)
A1	1.05	0.506	0.297	1.717	1.49	−25.80	7.69	0.509	13.25
A2	0.79	0.367	0.20	4.515	2	−25.26	7.96	0.531	14.82
A3	0.615	0.260	0.126	1.05	11.37	7.13	24.16	0.524	16.23
B1	1.22	0.64	0.0425	9.25	1.73	−28.48	6.35	0.493	22.84
B2	0.908	0.460	0.305	2.2	2.11	−22.11	9.54	0.50	24.46
B3	0.708	0.340	0.197	6.4	1.65	−25.42	7.88	0.493	25.93
C1	**1.13**	**0.645**	**0.436**	**10.2**	**29.35**	**23.60**	**32.40**	**0.297**	**90.02**
C2	0.868	0.464	0.279	1.79	1.50	−31.22	4.987	0.353	82.88
C3	0.678	0.341	0.201	5.3	2.02	−23.04	9.07	0.385	78.60
GAA FET [28]	0.5	3.2	2.3	1.2	2.1	Not Given	Not Given	Not Given	Not given

**Table 4:** Harmonic distortion analysis of all developed devices. Significant values are in bold.

Device	HD2 (dBm)	HD3 (dBm)	THD (dBm)	IMD3 (dBm)
A1	−9	−15.78	18.16613	89.80051
A2	−7	−18.51	19.78939	98.2727
A3	−6.24	−48.71	49.10806	180.81398
B1	−10.18	−13.09	16.58254	251.80033
B2	−3.47	−65.18	65.2723	108.39379
B3	−4.62	−16.15	16.79782	87.98462
C1	**−1.345**	**−19.47**	**19.5164**	**63.80899**
C2	−3.09	−13.41	13.7614	89.06302
C3	−12.44	−18.54	22.32678	98.87791
Si FET [29]	85	156.6	177.7	Not Given

## Data Availability

The datasets used and analyzed during the current study are available from the corresponding author on reasonable request.
